# Safety and feasibility of nucleus accumbens stimulation in five patients with epilepsy

**DOI:** 10.1007/s00415-014-7364-1

**Published:** 2014-05-07

**Authors:** Friedhelm C. Schmitt, Juergen Voges, Hans-Jochen Heinze, Tino Zaehle, Martin Holtkamp, Alexander B. Kowski

**Affiliations:** 1 Department of Neurology, University of Magdeburg, Leipzigerstr. 44, 39120 Magdeburg, Germany; 2 Department of Stereotactic Neurosurgery, University of Magdeburg, Magdeburg, Germany; 3 Leibniz Institute for Neurobiology, Magdeburg, Germany; 4 Department of Neurology, Epilepsy-Center Berlin-Brandenburg, Charité-Universitätsmedizin Berlin, Berlin, Germany

**Keywords:** Deep brain stimulation, Neuropsychology, Psychiatry, Quality of life, Seizure frequency, Seizure severity

## Abstract

In five adult patients with intractable partial epilepsy, safety and feasibility of chronic bilateral electrical stimulation of the nucleus accumbens (NAC) were assessed, also providing initial indications of therapeutic efficacy. Concurrent medication remained unchanged. In this phase 1 trial, clinical outcome parameters of interest were Quality of Life in Epilepsy questionnaire (QOLIE-31-P), Beck Depression Inventory, Mini International Neuropsychiatric Interview, neuropsychological testing, and Liverpool Seizure Severity Scale. Those data were obtained after 6 months of NAC stimulation and compared to the equivalent assessments made directly before implantation of electrodes. Additionally, monthly frequencies of simple partial seizures, complex partial seizures (CPS), and generalised tonic–clonic seizures (GTCS) were assessed during 3 months before electrode implantation and at the end of 6-month NAC stimulation. Proportion of responders, i.e. ≥50 % reduction in frequency of disabling seizures (sum of CPS and GTCS), was calculated. Main findings were unchanged psychiatric and neuropsychological assessment and a significant decrease in seizure severity (*p* = 0.043). QOLIE-31-P total score trended towards improvement (*p* = 0.068). Two out of five participants were responders. The median reduction in frequency of disabling seizures was 37.5 %. In summary, we provide initial evidence for safety and feasibility of chronic electrical stimulation of the NAC in patients with intractable partial epilepsy, as indicated by largely unchanged neurocognitive function and psychiatric comorbidity. Even though our data are underpowered to reliably assess efficacy, the significant decrease in seizure severity provides an initial indication of antiictal efficacy of NAC stimulation. This calls for larger and at best randomised trials to further elucidate efficacy of NAC stimulation in patients with pharmacologically intractable epilepsy.

## Introduction

In patients with intractable partial epilepsies, optimal adjustment of antiepileptic drugs (AED) fails to improve seizure frequency in at least 65 % of patients [1]. Presurgical evaluation of these patients leaves a substantial proportion of an estimated 40 % unresected [2], either due to ineligibility for surgery or because patients decide against this non-reversible but potentially curative procedure [3]. This difficult-to-treat patient group needs alternative treatment options. In the last decade, deep brain stimulation (DBS) has received increasing interest as a therapeutic option for these patients [4, 5]. Even though DBS is reversible and verifiably safe [6], so far this procedure has not been broadly used for intractable epilepsy. This may be partly due to the fact that ––in contrast to most resective treatment strategies––DBS is not a causative treatment approach and rarely results in seizure freedom [7]. Also, in patients treated with DBS for indications other than epilepsy, cognitive [8, 9], psychiatric [8, 10, 11], or behavioural side effects [12, 13] have been known for decades and might overshadow a differential view on this new treatment strategy for particular epilepsy patients.

Recently, a randomised controlled clinical trial demonstrated efficacy of electrical stimulation of the anterior nucleus of the thalamus (ANT) in terms of seizure frequency reduction [14]. In particular, patients with temporal lobe epilepsy appear to benefit from ANT stimulation [14, 15]. However, depression and memory impairments were significantly increased in patients with ANT stimulation [14]. Another single-blind trial evaluated the antiictal efficacy of centromedian thalamic (CMT) nucleus DBS [16]. The authors found no relevant effect in terms of seizure frequency reduction in patients with frontal lobe epilepsy, whereas in patients with generalised epilepsy, mean reduction in seizure frequency was 77 %. Other targets, such as the Ncl. subthalamicus have been discussed as possibly effective in small uncontrolled trials [17–19]. Recently, this target has been chosen for distinct epilepsy syndromes, such as progressive myoclonus epilepsy [18] and epilepsy due to ring chromosome 20 [20].

To identify and characterise new DBS targets for patients with difficult-to-treat epilepsy, we assessed safety and indications of chronic bilateral stimulation of the nucleus accumbens (NAC).

This structure plays a decisive role in both functional and anatomical connectivity between frontal and temporal lobes [21, 22]. In rodent models of generalised [23, 24] and both temporal [25–28] and frontal lobe seizures [29], the NAC has been shown to be involved in propagation of epileptic activity. Furthermore, NAC stimulation has been demonstrated to elicit euphoria in psychiatrically unaffected patients [30] and to ameliorate symptoms in patients with major depression [31]. Patients with epilepsy are at high risk for comorbid depression [32] and common network characteristics for both disorders have been proposed [32, 33].

In the following, we report clinical outcome of NAC stimulation in five patients with intractable partial epilepsy following an in-house protocol. We assessed the effect of electrical stimulation on quality of life, psychiatric and neuropsychological parameters, and additionally on severity and frequency of disabling seizures.

## Methods

### In-house protocol for deep brain stimulation

Five patients with medically intractable partial epilepsy (defined as failure of at least two AEDs in adequate doses to produce seizure freedom for at least 12 months [34]) became part of our in-house protocol for bilateral DBS of the NAC between January 2010 and December 2012. All patients first underwent comprehensive assessment for potential resective epilepsy surgery. Resection or advised invasive EEG monitoring was refused by the patients, or resection was not advisable at all or had been ineffective. Subsequently, patients were only offered DBS surgery if they had an average frequency of at least one disabling seizure per month (complex partial seizure [CPS], and/or generalised tonic–clonic seizure [GTCS]). Patients were not offered DBS if they suffered from progressive neurodegenerative disorders or had additional non-epileptic seizures, if IQ was lower than 70, if they were pregnant, or if they were currently treated with vagus nerve stimulation.

As chronic stimulation of the vagus nerve and of the ANT has been shown to be efficacious in large-scale controlled trials [14, 35], all patients with intractable and non-resectable partial epilepsy were subsequently offered these widely accepted minimally invasive surgery options and our in-house protocol for NAC stimulation (details see below). The five patients reported here took part in pre-operation interviews, in which all eligible patients were informed about different levels of evidence concerning safety and efficacy, current legal status of approval, and the extent of invasiveness of each surgical procedure. The possible risk of any surgical intervention led ten eligible patients to decide against any optional minimally invasive procedure. Two patients opted for VNS because the surgical intervention does not involve the brain, and another two patients decided for DBS of the ANT because of the greater evidence for efficacy. According to our pre-interventional discussions with patients, those who elected to undergo DBS felt that the risk of invasive surgery was outweighed by the possible benefit of direct effects on deep brain structures, as opposed to an indirect influence via a cranial nerve.

Outcome parameters of interest were mean change in patient-reported outcome questionnaires including Liverpool Seizure Severity Scale (LSSS) [36], Beck Depression Inventory (BDI-IA) [37], Quality of Life in Epilepsy Questionnaire (QOLIE-31-P) [38], and a standardised psychiatric interview employing Mini International Neuropsychiatric Interview (MINI) [39], as well as a neuropsychological test battery. The latter covered in detail the areas of attentional performance [test of attentional performance (TAP 2.2)], cognitive speed [trail making test (TMT), performance evaluation system subtest 7 (LPS, i.e. Leistungspruefungssystem, subtest 7), d2–attention stress test], executive function [“Regensburger” word fluency test (RWT), Hamasch 5-point test [H5PT)], memory and learning functions [verbal learn and memory test (VLMT), Wechsler Memory Scale-Revised (WMS-R)], and word retrieval (Boston naming test). All parameters were preoperatively assessed and compared to the results at the end of 6-month NAC stimulation. We also assessed the proportion of responders (≥50 % seizure frequency reduction) comparing the mean frequency of disabling seizures surveyed in the 3 months prior to electrode implantation to the data after 6 months of NAC stimulation. AEDs remained unchanged 3 months before electrode implantation and during 6 months of follow-up.

### Surgery

**Fig. 1:**
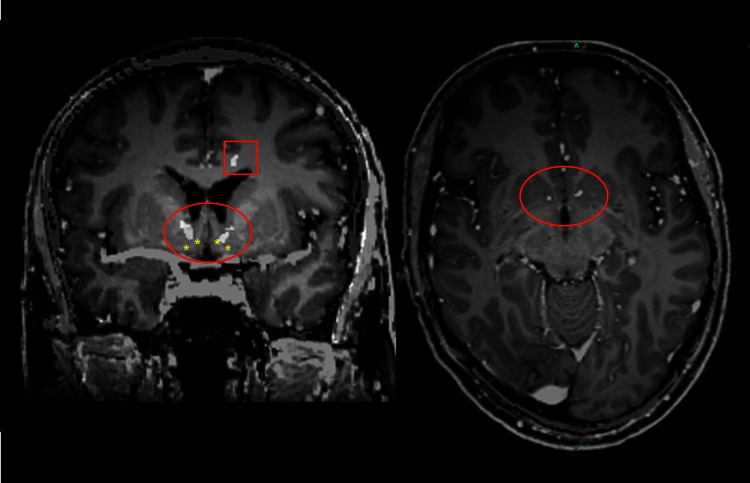
Distal electrode position in the NAC, depicted postoperatively with CT-fused presurgical T1–MRI in coronal and axial view (*red circles*). Both distal contacts of the quadripolar electrodes were placed in the NAC as intended, the third contact within the transition area to the medial border of the abutting internal capsule and the highest, i.e. the fourth contact at a point in the most medial part of the capsule or in the transition area to the caudate. *Asterisks* mark the medial and ventral border of the NAC region. The *red square* depicts an electrode lead to the left anterior nucleus of thalamus

Implantation of DBS systems was performed under general anaesthesia. Using standardised stereotactic technique, four Medtronic Model 3387 DBS leads (Medtronic^®^, Minneapolis, MN, USA) were implanted bilaterally into the NAC and ANT and subsequently connected to a single impulse generator (IPG; Activa-PC, Medtronic^®^, MA, USA). We defined the NAC target referred to the most distal contact of the quadripolar electrode to a point 2 mm rostral to the anterior commissure at the level of the mid-sagittal plane, 3–4 mm ventral and 6–8 mm lateral of the midline [40]. In addition, we modified the atlas coordinates according to the individual treatment planning-MRI displaying on coronal reconstructions of a 3D-MPRAGE series (3 T scanner, 0.8 mm^3^ resolution) the medial border (vertical limb of Broca’s diagonal band) and the ventral border (olfactory tubercle/horizontal limb of Broca’s diagonal band) of the NAC region (Fig. 1). The angulation of the trajectory relative to the intercommissural line was chosen to cover with two electrode contacts the central/lateral part (NAC core) and the medial part (NAC shell) [40, 41]. ANT targeting was also guided by atlas-based coordinates (Schaltenbrand and Wahren, 6 mm lateral to, 8 mm anterior to and 12 mm above the midcommisural point) and direct visualisation of this nucleus on 3D-MPRAGE images [42]. The electrode localisation was documented intraoperatively by stereotactic X-ray imaging using X-ray tubes installed in the OR and by postoperative CT examinations. CT as well as X-ray images were fused with the planning-MRI (Fig. 1).

Following surgery, NAC stimulation was initiated after mean 20.4 days (±10.5) and continued for 6 months (125 Hz, 5 V, 90 µs, 1 min on, 5 min off). Voltage reduction in 2 V steps was allowed in case of unexpected events such as pending series of disabling seizures, status epilepticus, or severe cognitive or psychiatric impairments. Additional ANT electrode implantation was chosen to allow for alternative chronic stimulation of the ANT in case of ineffective NAC stimulation (but in all five patients, ANT stimulation has not been used). Chronic stimulation of the ANT has been shown to be effective regarding frequency of CPS and severely impairing seizures [14]. The Institutional Review Board of the University of Magdeburg approved the surgical procedure (registration number 03/08). All patients granted written informed consent for implantation of the neurostimulation system.

### Statistics

Due to interindividual heterogeneity, scores of neurocognitive testing at the end of the NAC stimulation period were related to values collected prior to electrode implantation and expressed as fractions of one. After testing for Gaussian distribution (Kolmogorov–Smirnov test), which was negative for all parameters, the Wilcoxon rank sum test was used for comparison of ordinal data. Categorial data were tested with Fisher’s exact test. *P* values <0.05 (two-sided) were considered statistically significant.

## Results

Five patients (2 females), 30–53 years old (mean, 41.6 ± 10.6 years), were diagnosed with epilepsy 17–32 years (mean 23.6 ± 7.3 years) prior to DBS surgery. Clinical details are summarised in Table 1. Presurgical video-EEG evaluation revealed seizure onset zones in left mesio-temporal (2 patients), bilateral mesio-temporal, left fronto-temporal or bilateral frontal (each 1 patient) structures. Only one patient with left mesio-temporal epilepsy had undergone prior resective surgery (anterior temporal lobectomy due to hippocampal sclerosis, no seizure freedom), all other patients had non-lesional 1.5 or 3.0 T MRI. At time of electrode implantation, patients were administered 2–4 (mean 2.6) AEDs.

**Table 1:** Clinical data

Participant	Sex	Age (years)	Epilepsy duration (years)	Region of seizure onset	MRI	Seizures during study	AEDs during study
1	Male	30	18	Bilateral frontal	Normal	CPS, GTCS	PHT, LTG, ZNS, LCM
2	Female	53	20	Bilateral mesio-temporal	Normal	CPS, GTCS	LCM, LTG
3^a^	Male	40	31	Left mesio-temporal	Hippocampal sclerosis	SPS, GTCS	LCM, LTG
4	Male	33	32	Bilateral frontal and left mesio-temporal	Normal	SPS, GTCS	STP, OXC, CLB
5	Female	52	17	Left mesio-temporal	Normal	SPS, CPS, GTCS	LTG, LCM

*MRI* magnet resonance imaging, *SPS* simple partial seizures, *CPS* complex partial seizures, *GTCS* generalised tonic–clonic seizures, *AEDs*, antiepileptic drugs, *PHT* phenytoin, *LTG* lamotrigine, *ZNS* zonisamide, *LCM* lacosamide, *STP* stiripentole, *OXC* oxcarbazepine, *CLB* clobazam

^a^Ineffective epilepsy surgery

Regarding seizure severity, the LSSS was significantly reduced after 6 months of NAC stimulation as compared to scores prior to implantation of electrodes (*p* = 0.043).The QOLIE-31-P total score as well as its subscales “seizure worry” and “overall” improved only by trend (both *p* = 0.068). Neuropsychological testing, the MINI and the BDI-IA did not show significant differences after NAC stimulation in comparison to examinations before electrode implantation (Table 2). Two out of five patients were responders with ≥50 % frequency reduction of disabling seizures (patient #3, 50 %, and patient #4, 85.4 % reduction). Both responders’ and non-responders’ distal contacts were inside the target zone, as postoperative CT as well as X-ray images fused with the planning-MRI have revealed.

**Table 2:** Clinical outcome after six months of NAC stimulation

	Prior to electrode implantation	After NAC stimulation	Wilcoxon test	Fisher’s exact test
Patients reported outcome questionnaires (mean ± SD)				
LSSS	59.00 ± 3.67	49.60 ± 8.41	**0.043**	
BDI-1A	10.80 ± 7.46	6.60 ± 6.07	0.104	
QOLIE-31-P-total score	45.87 ± 09.82	50.72 ± 08.32	0.068	
Subscale “energy”	23.75 ± 20.67	14.69 ± 03.87	0.581	
Subscale “mood”	25.25 ± 12.02	31.90 ± 31.05	1.000	
Subscale “daily activities”	13.60 ± 15.16	30.00 ± 31.74	0.144	
Subscale “cognition”	16.38 ± 13.88	26.71 ± 23.14	0.465	
Subscale “medication effects”	34.17 ± 36.27	38.17 ± 16.30	0.465	
Subscale “seizure worry”	04.85 ± 03.52	19.30 ± 14.60	0.068	
Subscale “overall”	20.00 ± 17.80	30.06 ± 14.27	0.068	
Neuropsychiatric interview—MINI (number of participants)				
Major depression	2/5	1/5		1.000
Suicidal tendency	1/5	1/5		1.000
Mania	0/5	0/5		n.c.
Panic disturbance	1/5	0/5		1.000
Agoraphobia	0/5	0/5		n.c.
Social phobia	0/5	0/5		n.c.
Obsessive–compulsive disorder	0/5	0/5		n.c.
Substance addiction	0/5	0/5		n.c.
Psychosis	0/5	0/5		n.c.
Generalised anxiety disorder	0/5	2/5		0.444
Neurocognition^d^	1	1.06 ± 0.18	0.225	
Attentional performance	1	*1.08* ± *0.18*	*0.465*	
Cognitive speed	1	0.97 ± 0.05	0.225	
Executive functions	1	1.29 ± 0.47	0.255	
Memory and learning function	1	0.95 ± 0.30	0.500	
Word retrieval	1	1.03 ± 0.04	0.258	
Frequency of seizure types in 28-day periods (mean ± SD)				
SPS^a^	0.27 ± 0.37	0.50 ± 0.87	0.593	
CPS^b^	1.27 ± 1.64	2.20 ± 4.01	1.000	
GTCS^c^	2.27 ± 3.23	1.40 ± 1.04	0.893	
Disabling seizures	3.53 ± 2.79	3.60 ± 4.93	0.893	

*SD* standard deviation, *n.c* not calculated, *NAC* nucleus accumbens, *SPS* simple partial seizure, *CPS* complex partial seizure, *GTCS* generalised tonic–clonic seizure, *LSSS* Liverpool Seizure Severity Score, *BDI-1A* Beck Depression Inventory, version IA, *QOLIE-31-P* Quality of Life in Epilepsy, *M.I.N.I* Mini International Neuropsychiatric Interview

^a^3 out of 5 patients with SPS

^b^3/7 CPS

^c^All experienced GTCS

^d^Due to interindividual heterogeneity, results of neurocognitive testing after NAC stimulation were related to the individual values prior to electrode implantation and expressed as fractions of 1 (±SD)

**Fig. 2:**
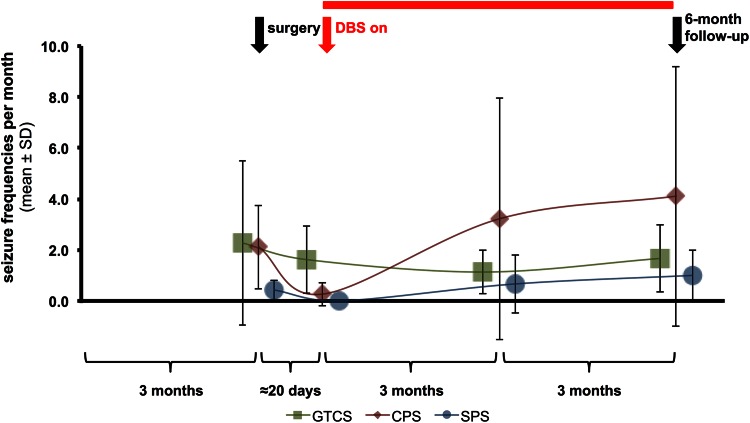
Time course of mean frequency of simple partial (SPS), complex partial (CPS) and generalised tonic–clonic seizures (GTCS). Following 3 months of seizure survey before electrode implantation (*black arrow*, surgery), stimulation of the nucleus accumbens (NAC) was started 22.4 days (±10.5) days post-surgery (*red arrow*, DBS on). Frequency of all seizure types was assessed 3 and 6 months after onset of NAC stimulation (*black arrow*, 6-month follow-up). After 6 months of NAC stimulation, frequency of CPS has increased, whereas that of GTCS has decreased. DBS: deep brain stimulation

Overall, we found a median reduction of disabling seizure frequency of 37.5 %, but no significant changes in the mean frequencies of simple partial seizures (SPS), CPS and GTCS (Table 2). The time course of mean frequency of SPS, CPS and GTCS is depicted in Fig. 2.

BDI-IA and/or MINI revealed depressive symptoms in three patients (#3, #4, #5) prior to electrode implantation. Following NAC stimulation, depressive symptoms resolved in one of them (patient #4). Patients #1 and #2 did not have depressive symptoms before surgery and at the end of NAC stimulation. With regard to other psychiatric entities assessed by MINI, patients #3 and #5 had new onset generalised anxiety disorder after 6-month NAC stimulation. However, only patient #5 had an increase in the total number of psychiatric conditions (from 0 to 2 [generalised anxiety and major depression]). In this patient, stimulation voltage had to be reduced as a precaution from 5 to 3 V in accordance with our in-house protocol due to abrupt increase in seizure frequency.

## Discussion

In five patients with intractable partial epilepsy, clinical consequences of 6-month NAC stimulation were comprehensively assessed including quality of life, and psychiatric and neuropsychological signs or symptoms, and seizure severity. The main finding was a lack of deterioration of neuropsychological parameters or psychiatric conditions, and overall reduction in subjectively perceived seizure severity. Concerning seizure frequency, our small cohort revealed a median frequency reduction of disabling seizures of 37.5 %, two out of five patients experienced a seizure reduction of at least 50 %. Looking at the time course of seizure frequency postsurgically, there was a decrease before stimulation was initiated (Fig. 2). This is similar to the observation by Fisher et al. [14] and Hodaie et al. [42] after insertion of electrodes for ANT stimulation, and has also been reported by Valentin et al. [16] after electrode implantation into CMT. Whether this pre-stimulation decrease in seizure frequency suggests a short-term placebo effect or a microlesional effect by electrode implantation is still a matter of discussion.

The SANTE (stimulation of the anterior nuclei of thalamus for epilepsy) trial, so far the only large-scale randomised controlled DBS study in epilepsy, showed that ANT stimulation results in significant seizure frequency reduction after 3 months [14]. Complementary to the 110 patients reported in SANTE, our five patients showed significantly reduced seizure severity after 6 months of NAC stimulation in spite of an overall unchanged mean seizure frequency. The non-significant decrease in frequency of GTCS and increase of CPS might be an explanation for this significant reduction in seizure severity. The trend to less GTCS may also be reflected by the decrease in “seizure worry” score in the quality-of-life assessment. Also, the item “seizure worry” correlated with seizure severity, as found in other studies [43]. Thus, we tend to interpret the non-significant trend to reduction in “seizure severity” as plausible. The lack of statistical significance with regard to improvement of this item and the summarised quality-of-life score is likely due to the small number of patients. Similar to our study, Valentin et al. comprehensively assessed the effect of DBS of the CMT nucleus in a small number of patients (*n* = 11; frontal lobe and generalised epilepsies) [16]. In seven of their patients, seizure severity score and quality of life assessments were available. Compared to the results before implantation, a significant improvement both in seizure severity and QOLIE-32-P total score was noted after 3 months of CM stimulation. Thus, they found that quality-of-life score improves parallel to reduction of seizure severity score. This correlation has already been validated in large cohorts and seems to be independent from seizure frequency [43–45].

NAC stimulation has been shown to ameliorate symptoms in patients with major depression [31, 46]. Our standardised psychiatric evaluation by MINI revealed no significant change in our cohort. However, one patient benefited from NAC stimulation concerning depressive symptoms. It cannot be determined whether this observation is seizure- or target-dependent, because this patient (#4) was a responder with an 85.4 % reduction in seizure frequency. Worsening of cognition and depressive symptoms as reported due to ANT stimulation in the SANTE trial, however, are probably target-dependent. Even though seizure frequency declined after ANT stimulation, patients complained significantly more often of depressive symptoms and memory deficits than unstimulated control subjects. In one of our patients (#4), depressive symptoms resolved with decreased seizure frequency, and patient #5 experienced an increase both in seizure frequency and in numbers of psychiatric symptoms. Both observations may suggest a seizure-dependent effect on psychiatric symptoms. We therefore cannot report an independent antidepressant effect of NAC stimulation in our cohort.

Interestingly, our overall cohort did not show changes in neuropsychological subscores (Table 2), indicating that NAC stimulation seems to be inert concerning cognitive function. An open observational study on ANT stimulation, which allowed for AED changes, reported amelioration in verbal memory during 12 months of stimulation [47]. In the controlled SANTE trial, however, significantly more patients complained of memory deficits and similarly of depressive symptoms [14]. A plausible explanation for the observed neurocognitive side effects is the well-known involvement of the ANT in memory processing [48, 49].

The major methodological limitation of this case series is the small number of patients, which renders our findings primarily anecdotal. Also, a placebo effect of high expectation due to an invasive intervention cannot be excluded due to the uncontrolled and unblinded protocol. This, however, included a long time period of 6 months, which in our view makes a pure placebo effect rather unlikely. Furthermore, placebo studies found that a positive response due to elevated post-interventional expectancy is negligible for a long-term placebo effect [50]. Lastly, due to the unblinded in-house protocol we cannot exclude that microlesional effects of the NAC or the ancillary implanted ANT electrodes are in part responsible for the significantly reduced seizure severity score. However, the SANTE trial showed normalisation of seizure frequency within 3 months in the cohort of unstimulated patients [14]. In our cohort, there was a marked postsurgical seizure frequency reduction before NAC stimulation onset, which was not sustained for more than a month (Fig. 2). These two observations suggest a subsidence of a putative microlesional effect at the latest within 3 months.

To reliably assess efficacy of NAC stimulation, the cohort is underpowered compared to well-established minimally invasive procedures such VNS and DBS for ANT. However, initial evidence for safety and feasibility of NAC stimulation in patients with intractable partial epilepsy is presented, and the findings may point to some preliminary evidence for antiictal efficacy. The lack of neurocognitive and psychiatric side effects merits possible advantages for the target NAC, so we conclude that further exploration of this target would be worthwhile. To assess for placebo or microlesional effects, a randomised controlled trial would be required. Furthermore, a larger number of patients would be necessary to confirm antiictal effects of NAC stimulation and to discriminate putative responders and non-responders according to their electroclinical features.

## References

[1] Luciano AL, Shorvon SD (2007). Results of treatment changes in patients with apparently drug-resistant chronic epilepsy. Ann Neurol.

[2] Picot M-C, Baldy-Moulinier M, Daurès J-P (2008). The prevalence of epilepsy and pharmacoresistant epilepsy in adults: a population-based study in a Western European country. Epilepsia.

[3] Bien CG, Raabe AL, Schramm J (2013). Trends in presurgical evaluation and surgical treatment of epilepsy at one centre from 1988-2009. J Neurol Neurosurg Psychiatry.

[4] Theodore WH, Fisher RS (2004). Brain stimulation for epilepsy. Lancet Neurol.

[5] Boon P, Raedt R, de Herdt V (2009). Electrical stimulation for the treatment of epilepsy. Neurother J Am Soc Exp Neurother.

[6] Voges J, Waerzeggers Y, Maarouf M (2006). Deep-brain stimulation: long-term analysis of complications caused by hardware and surgery––experiences from a single centre. J Neurol Neurosurg Psychiatry.

[7] Hariz M, Blomstedt P, Zrinzo L (2013). Future of brain stimulation: new targets, new indications, new technology. Mov Disord Off J Mov Disord Soc.

[8] Voon V, Kubu C, Krack P (2006). Deep brain stimulation: neuropsychological and neuropsychiatric issues. Mov Disord.

[9] Kim H-J, Jeon BS, Yun JY (2013). Initial cognitive dip after subthalamic deep brain stimulation in Parkinson disease. J Neurol.

[10] Appleby BS, Duggan PS, Regenberg A (2007). Psychiatric and neuropsychiatric adverse events associated with deep brain stimulation: a meta-analysis of ten years’ experience. Mov Disord.

[11] Shapira NA, Okun MS, Wint D (2006). Panic and fear induced by deep brain stimulation. J Neurol Neurosurg Psychiatry.

[12] Visser-Vandewalle V, Temel Y, Boon P (2003). Chronic bilateral thalamic stimulation: a new therapeutic approach in intractable Tourette syndrome. Report of three cases. J Neurosurg.

[13] Lulé D, Heimrath J, Pinkhardt EH (2012). Deep brain stimulation and behavioural changes: is comedication the most important factor?. Neurodegener Dis.

[14] Fisher R, Salanova V, Witt T (2010). Electrical stimulation of the anterior nucleus of thalamus for treatment of refractory epilepsy. Epilepsia.

[15] Zumsteg D, Lozano AM, Wennberg RA (2006). Mesial temporal inhibition in a patient with deep brain stimulation of the anterior thalamus for epilepsy. Epilepsia.

[16] Valentín A, García Navarrete E, Chelvarajah R (2013). Deep brain stimulation of the centromedian thalamic nucleus for the treatment of generalized and frontal epilepsies. Epilepsia.

[17] Chabardès S, Kahane P, Minotti L (2002). Deep brain stimulation in epilepsy with particular reference to the subthalamic nucleus. Epileptic Disord.

[18] Wille C, Steinhoff BJ, Altenmüller D-M (2011). Chronic high-frequency deep-brain stimulation in progressive myoclonic epilepsy in adulthood––report of five cases. Epilepsia.

[19] Loddenkemper T, Pan A, Neme S (2001). Deep brain stimulation in epilepsy. J Clin Neurophysiol.

[20] Jobst B (2010). Brain stimulation for surgical epilepsy. Epilepsy Res.

[21] Sturm V, Lenartz D, Koulousakis A (2003). The nucleus accumbens: a target for deep brain stimulation in obsessive-compulsive- and anxiety-disorders. J Chem Neuroanat.

[22] van Kuyck K, Gabriëls L, Cosyns P, Sakas DE, Simpson BA (2007). Behavioural and physiological effects of electrical stimulation in the nucleus accumbens: a review. Operative neuromodulation.

[23] Riban V, Pereira de Vasconcelos A, Phâm-Lê BT (2004). Modifications of local cerebral glucose utilization in thalamic structures following injection of a dopaminergic agonist in the nucleus accumbens—involvement in antiepileptic effects?. Exp Neurol.

[24] Deransart C, Riban V, Lê B (2000). Dopamine in the striatum modulates seizures in a genetic model of absence epilepsy in the rat. Neuroscience.

[25] Pereira de Vasconcelos A, Mazarati AM, Wasterlain CG (1999). Self-sustaining status epilepticus after a brief electrical stimulation of the perforant path: a 2-deoxyglucose study. Brain Res.

[26] Löscher W, Ebert U, Lehmann H (1996). Kindling induces a lasting, regionally selective increase of kynurenic acid in the nucleus accumbens. Brain Res.

[27] Wahnschaffe U, Loescher W (1991). Anticonvulsant effects of ipsilateral but not contralateral microinjections of the dopamine D2 agonist LY 171555 into the nucleus accumbens of amygdala-kindled rats. Brain Res.

[28] Lothman EW, Hatlelid JM, Zorumski CF (1985). Functional mapping of limbic seizures originating in the hippocampus: a combined 2-deoxyglucose and electrophysiologic study. Brain Res.

[29] Ma J, Leung LS (2010). Kindled seizure in the prefrontal cortex activated behavioral hyperactivity and increase in accumbens gamma oscillations through the hippocampus. Behav Brain Res.

[30] Okun MS, Bowers D, Springer U (2004). What’s in a “smile?” Intra-operative observations of contralateral smiles induced by deep brain stimulation. Neurocase.

[31] Schlaepfer TE, Bewernick BH (2013). Deep brain stimulation for major depression. Handb Clin Neurol.

[32] Kanner AM (2013). The treatment of depressive disorders in epilepsy: what all neurologists should know. Epilepsia.

[33] Valente KDR, Busatto Filho G (2013). Depression and temporal lobe epilepsy represent an epiphenomenon sharing similar neural networks: clinical and brain structural evidences. Arq Neuropsiquiatr.

[34] Kwan P, Arzimanoglou A, Berg AT (2010). Definition of drug resistant epilepsy: consensus proposal by the ad hoc Task Force of the ILAE Commission on Therapeutic Strategies. Epilepsia.

[35] Handforth A, DeGiorgio CM, Schachter SC (1998). Vagus nerve stimulation therapy for partial-onset seizures: a randomized active-control trial. Neurology.

[36] Scott-Lennox J, Bryant-Comstock L, Lennox R (2001). Reliability, validity and responsiveness of a revised scoring system for the Liverpool Seizure Severity Scale. Epilepsy Res.

[37] Byrne BM, Baron P, Larsson B (1995). The Beck Depression Inventory: testing and cross-validating a second-order factorial structure for Swedish nonclinical adolescents. Behav Res Ther.

[38] Cramer JA, Arrigo C, Van Hammée G, Bromfield EB (2000). Comparison between the QOLIE-31 and derived QOLIE-10 in a clinical trial of levetiracetam. Epilepsy Res.

[39] Sheehan DV, Lecrubier Y, Sheehan KH (1998). The Mini-International Neuropsychiatric Interview (M.I.N.I.): the development and validation of a structured diagnostic psychiatric interview for DSM-IV and ICD-10. J Clin Psychiatry.

[40] Mai PG, Assheuer JK (2004). Atlas of the human brain.

[41] Voges J, Müller U, Bogerts B (2013). Deep brain stimulation surgery for alcohol addiction. World Neurosurg.

[42] Buentjen L, Kopitzki K, Schmitt FC (2013). Direct targeting of the thalamic anteroventral nucleus for deep brain stimulation by T(1)-weighted magnetic resonance imaging at 3 T. Stereotact Funct Neurosurg.

[43] Hodaie M, Wennberg RA, Dostrovsky JO, Lozano AM (2002). Chronic anterior thalamus stimulation for intractable epilepsy. Epilepsia.

[44] Harden CL, Maroof DA, Nikolov B (2007). The effect of seizure severity on quality of life in epilepsy. Epilepsy Behav.

[45] Jehi LE, O’Dwyer R, Najm I (2009). A longitudinal study of surgical outcome and its determinants following posterior cortex epilepsy surgery. Epilepsia.

[46] Bautista RED, Tannahill Glen E (2009). Seizure severity is associated with quality of life independent of seizure frequency. Epilepsy Behav.

[47] Schlaepfer TE, Cohen MX, Frick C (2008). Deep brain stimulation to reward circuitry alleviates anhedonia in refractory major depression. Neuropsychopharmacol Off Publ Am Coll Neuropsychopharmacol.

[48] Oh Y-S, Kim HJ, Lee KJ (2012). Cognitive improvement after long-term electrical stimulation of bilateral anterior thalamic nucleus in refractory epilepsy patients. Seizure J Br Epilepsy Assoc.

[49] Aggleton JP, O’Mara SM, Vann SD (2010). Hippocampal-anterior thalamic pathways for memory: uncovering a network of direct and indirect actions. Eur J Neurosci.

[50] Staudigl T, Zaehle T, Voges J (2012). Memory signals from the thalamus: early thalamocortical phase synchronization entrains gamma oscillations during long-term memory retrieval. Neuropsychologia.

[51] Hyland ME (2011). Motivation and placebos: do different mechanisms occur in different contexts?. Philos Trans R Soc B Biol Sci.

